# Neuroendocrine Neoplasms in Pregnancy: A Narrative Review of Clinical Challenges and Therapeutic Limitations in the Absence of Established Safe Treatments

**DOI:** 10.3390/jpm15070272

**Published:** 2025-06-25

**Authors:** Mauro Daniel Spina Donadio, Maria Cecília Mathias-Machado, Danielly Scaranello Nunes Santana, Renata D’Alpino Peixoto

**Affiliations:** 1 Oncoclínicas, São Paulo 04538-132, SP, Brazil; 2 Oncoclínicas, Salvador 40050-410, BH, Brazil; cissa.mathias@gmail.com; 3 GineCare, Jundiaí 13208-703, SP, Brazil; dany.fmj@terra.com.br; 4 Medical Oncology Department, BC Cancer Agency, Vancouver, BC V5Z 4E6, Canada; renatadalpino@gmail.com

**Keywords:** neuroendocrine neoplasia, pregnancy, maternal–fetal medicine

## Abstract

Cancer during pregnancy is a rare but complex clinical scenario that affects approximately 0.1% of pregnant individuals and is associated with increased maternal morbidity. With the trend of delayed childbearing, the incidence of pregnancy-associated cancers is expected to rise. Neuroendocrine neoplasms (NENs), although rare in pregnancy, present unique diagnostic and therapeutic challenges due to their hormonal activity, histological diversity, and limited data on management in the gestational context. **Objectives**: This manuscript reviews the current evidence on the diagnosis, staging, and management of NENs during pregnancy, focusing on maternal–fetal safety, therapeutic limitations, and multidisciplinary care strategies. **Methods**: A comprehensive narrative review was conducted using relevant case reports, retrospective studies, clinical guidelines, and expert consensus documents addressing cancer in pregnancy and NEN-specific management. **Results**: Pregnancy complicates the evaluation and treatment of NENs due to overlapping symptoms, contraindications to standard imaging and systemic therapies, and unreliable biomarkers such as chromogranin A and 5-HIAA. Most systemic therapies for NENs, including somatostatin analogs, tyrosine kinase inhibitors, and peptide receptor radionuclide therapy, are contraindicated or lack safety data in pregnancy. Surgical interventions and supportive care require careful planning. Decisions regarding pregnancy continuation or termination must be individualized and supported by a multidisciplinary team. **Conclusions**: The management of NENs during pregnancy demands a highly individualized approach, coordinated among oncology, maternal–fetal medicine, and supportive care teams. Given the paucity of robust data, future research is essential to establish evidence-based guidelines and improve outcomes for both mother and fetus.

## 1. The Impact of Cancer Concurrent with Pregnancy

Cancer is the second leading cause of death among reproductive-age women, affecting approximately 0.1% of pregnant individuals [1,2]. Although rare, its clinical impact is significant due to the need to balance maternal and fetal health, posing challenges in diagnosis and treatment, as well as the risk of adverse events such as intensive care unit (ICU) admission, bleeding, preterm birth, low birth weight, and pregnancy loss [1]. With increasing maternal age and delayed childbearing, the incidence of pregnancy-associated cancer is expected to rise [3].

The most common cancers diagnosed during pregnancy include breast, cervical, and ovarian cancers, as well as melanoma, colorectal cancer, lymphomas, and leukemias [4,5,6,7]. Overlapping symptoms between malignancy and normal pregnancy may delay diagnosis and treatment, potentially affecting prognosis [8].

Evidence on cancer outcomes during pregnancy is limited, though some studies suggest poorer prognosis compared to non-pregnant patients [9,10,11,12]. These findings are complicated by confounding factors such as hormonal changes, diagnostic delays, and modified treatment protocols due to concerns about fetal safety.

While transplacental transmission of cancer is rare, it has been reported in aggressive tumors like metastatic melanoma, lung cancer, and certain hematologic malignancies [7,13,14,15,16,17], underscoring the need for close monitoring in select cases.

Taken together, the management of cancer during pregnancy demands a multidisciplinary, patient-centric approach that carefully considers individualized treatment timelines and therapeutic strategies. In this review, we explore the specific challenges associated with diagnosing and treating neuroendocrine neoplasms (NENs) during pregnancy—a topic that remains markedly underreported in the literature.

## 2. Neuroendocrine Neoplasms: Biological Diversity and Clinical Implications

Neuroendocrine neoplasms are a heterogeneous group of tumors with neuroendocrine differentiation that can arise in any organ. A significant proportion of NEN patients experience endocrine imbalances due to increased secretion of amines or peptide hormones, which can impact both their quality of life and prognosis. These tumors secrete peptide hormones and present with a wide range of symptoms depending on the specific hormone secreted. They also vary greatly in their metastatic patterns [18,19].

Histologically, NENs are divided into two major types: well-differentiated neuroendocrine tumors (NETs) and poorly differentiated neuroendocrine carcinomas (NECs).

The classification system for NENs has been updated several times, with the most recent revision in the 2022 WHO Classification of Endocrine and Neuroendocrine Neoplasms [20]. Tumors that retain both the molecular and morphological characteristics of neuroendocrine cells and are well differentiated are classified as NETs. These are graded based on the Ki-67 index: G1 (<3%), G2 (3–20%), and G3 (>20%). In contrast, tumors with marked cellular atypia, abnormal molecular or genetic features, and preserved neuroendocrine marker expression are classified as poorly differentiated NECs [20].

In terms of epidemiology, NENs are more frequently diagnosed in women, with many cases presenting as localized disease and classified as G1 tumors. In the United States, the most common primary sites for NETs are the lung, small intestine, and rectum [21].

Between 2000 and 2018, the age-adjusted incidence rate of NETs per 100,000 persons increased from 4.90 to 8.19 (annual percentage change [APC], 3.40; 95% confidence interval [CI], 3.13–3.67), with the most significant increases observed in grade 1, localized stage, and appendiceal NETs. Over the same period, the age-adjusted mortality rate increased 3.1-fold (APC, 4.14; 95% CI, 3.14–5.15). The 1-, 5-, and 10-year relative survival rates for all NETs were 80.5%, 68.4%, and 63.5%, respectively [22].

From the Surveillance, Epidemiology, and End Results (SEER) 18 registry, 112,256 patients diagnosed with NETs were identified between 2000 and 2018. Among them, 59,195 (52.7%) were women. The lung was the most common primary site (22.7%), followed by the small intestine (17.4%) and rectum (16.0%). From 2000 to 2017, the incidence of G1 NETs showed the greatest increase, rising from 0.25 to 4.33 per 100,000 persons (APC, 18.93; 95% CI, 17.42–20.46) [21].

Despite the growing prevalence, data on NENs during pregnancy are virtually absent from the literature, representing a critical gap in both oncologic and obstetric research.

### 2.1. Neuroendocrine Neoplasms in Hereditary Syndromes and Pregnancy

Neuroendocrine neoplasms (NENs) can arise in the context of several hereditary syndromes, including multiple endocrine neoplasia types 1 and 4 (MEN1, MEN4), von Hippel–Lindau disease (VHL), neurofibromatosis type 1 (NF1), and tuberous sclerosis complex (TSC) [23], as described in Table 1. Although these syndromes account for approximately 10–20% of all NENs, data on pregnancy outcomes in affected women remain limited. Hormonal changes during pregnancy may influence tumor growth, and diagnosis can be delayed due to overlapping symptoms with pregnancy-related conditions [23].

The most comprehensive study on MEN1 and pregnancy, conducted in Tasmania, included 26 MEN1-positive women with a total of 96 pregnancies. The prevalence of NENs was relatively low. Five pregnancies occurred following successful insulinoma resection, and five involved nonfunctioning PanNENs, with no disease progression during or after pregnancy—except for one case of a postnatally identified pancreatic mass. Four pregnancies occurred in women with hypergastrinemia, none of whom required pharmacologic treatment [25,26,27,28,29]. Gastroesophageal reflux was reported in three pregnancies; two patients had normal serum gastrin levels, while gastrin status was unknown in the third. Notably, only one pregnancy (1.3%) in the cohort involved known FDG-avid pulmonary lesions. These lesions remained stable, required no antenatal intervention, and showed no progression during postnatal follow-up [29].

The clinical course of NENs during pregnancy in women with VHL has been explored in a study involving 52 mutation carriers, including 26 women. Findings suggested that pregnancy does not significantly influence the development or progression of pancreatic neuroendocrine tumors (PanNENs) in this population. Interestingly, pregnant women showed a lower age-adjusted rate of symptom development [36]. Despite this generally indolent course, the risk of PanNEN progression—particularly after multiple pregnancies—should not be entirely dismissed. Based on personal clinical observations, rare instances of tumor growth have been noted postpartum. Although progression is uncommon, especially in the absence of pre-existing lesions, MRI screening should ideally be performed close to conception. During pregnancy, additional abdominal MRI should be guided by clinical symptoms [36,37].

Notably, NF1 is associated with increased obstetric complications. A population-based study covering over 19 million pregnancies between 1988 and 2009 identified 1553 NF1-associated cases (prevalence: 0.008%), with significantly increased risks of gestational hypertension (adjusted odds ratio [AOR] 1.6; 95% CI: 1.2–2.0), preeclampsia (AOR 2.8; 95% CI: 2.3–3.4), intrauterine growth restriction (AOR 4.6; 95% CI: 3.7–5.6), preterm labor (AOR 1.6; 95% CI: 1.4–1.9), and cesarean delivery (AOR 2.0; 95% CI: 1.8–2.3). However, NF1 was not associated with increased maternal mortality or thromboembolic complications during hospitalization [33].

### 2.2. Thymic Neuroendocrine Neoplasms and Pregnancy

Thymic neuroendocrine neoplasms (TNENs) are exceedingly rare entities, accounting for approximately 2–5% of all primary thymic malignancies and fewer than 0.5% of NENs overall [38]. Their occurrence during pregnancy is extraordinarily uncommon, with available data confined to isolated case reports, limiting the development of standardized clinical guidance.

A notable case described in the literature involved a 26-year-old woman diagnosed with an adrenocorticotropic hormone (ACTH)-secreting thymic carcinoid tumor, which resulted in Cushing’s syndrome. Following curative-intent surgical resection and adjuvant therapy, the patient subsequently achieved two pregnancies. However, both gestations were marked by tumor recurrence. One pregnancy was notably complicated by preeclampsia and metabolic decompensation, necessitating preterm cesarean delivery at 34 weeks. Despite these challenges, both neonates were delivered in good health, and the patient achieved long-term survival of over 11 years post-diagnosis [39].

In a separate case, a woman with untreated ACTH-producing TNEN presented with active Cushing’s syndrome during pregnancy. Despite ongoing hormonal activity and the absence of oncologic intervention during gestation, she delivered a healthy term neonate without significant maternal or neonatal complications. This case is among the first to document a successful pregnancy outcome in the setting of uncontrolled hormonally active TNEN, underscoring the variability and potential resilience in such clinical scenarios [40].

## 3. Diagnostic Challenges and Clinical Limitations in Pregnancy

Identifying certain cancers during pregnancy can be challenging, as symptoms may be mistaken for normal physiological changes. Similarly, NENs are rare in pregnancy, and gestation introduces several limitations in both the diagnosis and management of these tumors.

Accurate diagnosis of the cancer type and stage using appropriate imaging modalities—such as ultrasound, chest X-ray, and mammography with abdominal shielding to minimize fetal exposure to ionizing radiation—is a critical component of cancer management during pregnancy [41,42,43].

Managing NENs during pregnancy is further complicated by the risks associated with diagnostic imaging and biopsy procedures, requiring a cautious approach to minimize fetal radiation exposure. Risk assessment and staging rely on pathology and TNM classification, often utilizing contrast-enhanced imaging and positron emission tomography–computed tomography (PET-CT) with radiolabeled somatostatin analogs (SSAs). However, these imaging methods are contraindicated during pregnancy and should be deferred until after delivery.

Whole-body diffusion-weighted MRI (WB-DWI/MRI) is a feasible and highly accurate method for cancer staging in pregnant women and is considered the standard radiologic approach for staging pregnant oncology patients. Compared to conventional imaging methods such as CT and ultrasound, WB-DWI/MRI offers significant advantages by improving lesion detection and staging accuracy without exposing patients to radiation. Therefore, WB-DWI/MRI is the preferred imaging modality for oncologic staging during pregnancy, potentially improving treatment outcomes [44]. Although evidence on the safety of gadolinium-based contrast agents during pregnancy is limited, animal studies have shown that these agents can cross the placenta [7,43]. Additionally, there is currently no reliable data on the safety of iodinated contrast agents in pregnancy.

Furthermore, normal pregnancy may elevate chromogranin A (CgA) levels, partially due to the placenta’s neuroendocrine activity, making this marker unreliable for monitoring NENs during pregnancy [45]. Similarly, 5-hydroxyindoleacetic acid (5-HIAA) levels have been reported to increase progressively throughout pregnancy and return to baseline postpartum. Therefore, in pregnant patients with carcinoid syndrome, 5-HIAA levels should be interpreted with caution—mild elevations without corresponding clinical symptoms may be attributable to pregnancy. Nevertheless, 5-HIAA levels measured shortly after delivery can serve as a reliable follow-up marker in patients with NENs [46].

Thus, accurate diagnosis and staging during gestation, along with the detection of functioning syndromes, require a nuanced approach that integrates clinical suspicion with non-invasive diagnostic modalities, while deferring high-risk procedures until postpartum whenever possible. The WB-DWI/MRI should be used as the preferred imaging modality for oncologic staging during pregnancy, and minor alterations in specific laboratory tests for the diagnosis of functioning syndromes should be interpreted with caution.

## 4. Therapeutic Considerations and the Role of Personalized Medicine

Therapeutic strategies for NENs have been comprehensively addressed in recent international guidelines, including those published by the European Neuroendocrine Tumor Society (ENETS) and the European Society for Medical Oncology (ESMO) [47,48,49,50,51,52,53,54,55]. These guidelines provide detailed recommendations for diagnosis, staging, and treatment tailored to tumor grade, site of origin, and disease burden. However, despite these advancements, the management of NENs during pregnancy remains a significantly underexplored area. Current guidelines do not provide specific recommendations for pregnant patients, and there is a notable lack of consensus or standardized approaches for managing these cases.

The limited availability of safety data for most anticancer therapies during pregnancy further complicates clinical decision-making [56]. Children exposed to maternal chemotherapy are more likely to experience small for gestational age status, preterm birth, and increased perinatal morbidity and mortality in the early weeks of life, as well as a higher risk of cardiovascular and metabolic diseases later in life [1]. In the context of NENs, the evidence base is particularly sparse, with much of the literature comprising isolated case reports, rather than large-scale studies or clinical trials [57,58,59,60,61,62,63,64,65,66,67,68,69,70]. This scarcity of robust data makes it challenging to assess the risks and benefits of different treatment options for both the mother and fetus, thereby necessitating a highly individualized and multidisciplinary approach to care.

Given these uncertainties, it is essential that pregnant patients diagnosed with NENs receive thorough and empathetic counseling about all available management options. These discussions should include the potential continuation or termination of pregnancy—whether due to patient preference or the clinical necessity of initiating teratogenic treatments that cannot be delayed. Such counseling must be delivered in a supportive, non-directive manner and involve oncologists, maternal–fetal medicine specialists, and, when appropriate, ethics consultants. Ultimately, the goal is to ensure that patients are fully informed and empowered to make decisions that align with their values, medical needs, and reproductive goals.

### 4.1. Pregnancy Termination

When cancer is diagnosed during pregnancy, a multidisciplinary team—often including oncologists, obstetricians, maternal–fetal medicine specialists, neonatologists, and mental health professionals—must navigate complex, individualized decisions. The central challenge is balancing maternal treatment needs with fetal safety, often amid limited evidence and emotionally difficult choices.

Pregnancy termination occurs in about 9% to 28% of cases, most often in the first trimester, when the fetus is most susceptible to teratogenic effects from treatment [1,71]. These decisions are influenced by numerous factors, including cancer type, stage, gestational age at diagnosis, urgency of treatment, and the patient’s values and reproductive goals.

Oncologists play a key role in presenting treatment options and explaining how pregnancy-related modifications—such as delayed therapy or alternative regimens—might affect both maternal prognosis and fetal risk. These conversations require clear medical guidance and compassionate communication.

Management must also consider potential fetal outcomes, including prematurity, growth restriction, or teratogenicity, depending on the timing and nature of treatment. A collaborative, patient-centered approach is essential to align clinical priorities with the individual’s preferences and achieve the best possible outcomes for both mother and child.

### 4.2. Locoregional Therapies

Surgery for cancer during pregnancy can be performed at any gestational stage, but decisions depend on tumor location, gestational age, and urgency. Tumors in accessible areas like the skin or limbs may be removed safely with minimal fetal risk. However, surgery usually requires careful coordination to protect both mother and fetus.

When possible, delaying surgery until fetal viability (around 24 weeks) is advised, as earlier procedures carry increased risks such as miscarriage, preterm labor, and fetal growth issues due to anesthesia and surgical stress [7,41,43]. Tumors near the pelvis or requiring invasive procedures pose greater risks, including hemorrhage and fetal distress.

If surgery is needed before viability, a multidisciplinary team—obstetricians, oncologists, anesthesiologists—must weigh the risks and benefits, considering tumor size, type, and metastatic potential. Surgical planning should be individualized to balance cancer treatment with pregnancy safety and maternal preferences.

Transcatheter arterial chemoembolization (TACE) should generally be avoided during pregnancy due to potential risks to the developing fetus. TACE involves the use of imaging techniques such as X-rays, which can expose the fetus to radiation and potentially cause harm. Additionally, the chemotherapy drugs delivered directly to the tumor may cross the placenta [72]. The limited evidence on the use of TACE during pregnancy is primarily in the context of hepatocellular carcinoma, and the general consensus is to defer treatment until after pregnancy [73].

Radiotherapy (RT) is generally avoided during pregnancy due to fetal risks [56]. In rare emergencies, such as spinal cord compression or brain metastases, RT may be used cautiously, with uterine shielding and the lowest effective dose [56,74,75,76]. Modified techniques and shielding further reduce fetal exposure.

Close coordination among radiation oncologists, medical physicists, oncologists, and obstetricians is essential. Guidelines from the American College of Radiology and International Consensus Panels offer protocols to minimize RT risks and guide its use during pregnancy [41,77].

### 4.3. Systemic Treatment

In general, chemotherapy should be avoided during the first trimester due to the increased risk of teratogenic effects, which include major congenital malformations, impaired organ function, spontaneous abortions, and fetal death [43,78,79,80,81]. While the use of chemotherapy during the second and third trimesters has not been associated with significant teratogenic effects, it may still pose maternal and fetal risks, including low birth weight, preterm labor, and intrauterine growth restriction [5,78,80,82,83,84]. The potential benefits and risks of chemotherapy for both the pregnant individual and the fetus must be carefully evaluated before initiating treatment. Delaying treatment until after fetal maturity, with careful follow-up to rule out disease progression, is a safe option for individuals diagnosed with early-stage cancers [85,86]. In some cases, particularly with advanced-stage disease requiring urgent chemotherapy in the first trimester, pregnancy termination may be considered. If the pregnant individual decides to continue the pregnancy, the potential teratogenic risks should be thoroughly discussed [5,7].

Systemic treatment for NENs, however, is primarily administered using somatostatin analogs, tyrosine kinase inhibitors, peptide receptor radionuclide therapy (PRRT), and chemotherapy [47,48,49,50,51,52,53,54]. Unfortunately, the main systemic treatment options are either not recommended or contraindicated according to the main health agencies, as their effects on the fetus are not fully known or are associated with the risk of malformations (Table 2).

It is possible to observe, as shown in Table 2, a generally more conservative approach of the EMA compared to the FDA regarding drug use in pregnancy. While the FDA occasionally permits use based on a risk–benefit analysis (especially with Category B or D drugs), the EMA often categorically contraindicates drugs, particularly those associated with reproductive toxicity or radiation. These regulatory perspectives serve as critical guides for clinicians in making informed decisions regarding the use of potentially teratogenic or harmful agents in pregnant patients.

Regarding the safety and feasibility of using SSAs during pregnancy, the available data are limited and primarily derived from studies involving patients with acromegaly. Although most reported cases are reassuring, uncertainty remains about the overall safety of SSAs during pregnancy. Conflicting case reports have described both adverse and favorable outcomes [103,104,105]. Evidence suggests that both extended-release and immediate-release formulations can cross the placental barrier. Short-acting octreotide, in particular, has been detected at low levels in umbilical cord serum, amniotic fluid, and neonatal serum [106]. In one study, two pregnant individuals with GEP-NENs were treated with lanreotide Autogel during pregnancy, both resulting in favorable maternal and fetal outcomes [107].

Supportive care during pregnancy includes symptom management for conditions such as hypergastrinemia or hypoglycemia, using appropriate medical interventions [50,108]. Pharmacologic therapies—such as proton pump inhibitors for gastrinomas and SSAs for various types of NENs—are employed under careful supervision, with the goal of controlling hormone secretion and tumor progression. Close monitoring of hormonal levels, fetal development, and maternal health is essential throughout treatment.

Carcinoid syndrome requires particular attention during pregnancy and delivery. Long-acting SSAs are considered a viable treatment option, especially during the second and third trimesters. While no specific clinical guidelines currently exist for the management of carcinoid crisis during labor and delivery, some case reports—and a recent consensus statement—recommend the use of octreotide. This involves an initial bolus dose followed by symptom-guided continuous infusion as needed [65,106,109,110].

In summary, personalized medicine plays a critical role in the therapeutic planning of NENs, enabling the customization of treatment strategies based on tumor grade, biological behavior, and maternal–fetal physiology. For instance, choosing between systemic therapy, surgery, or expectant management requires balancing oncologic urgency with the safest gestational window for intervention. Integrating genomic profiling and hormone receptor analysis can also inform the use of targeted therapies post-delivery, when safer systemic options become viable. In cases where aggressive treatment is unavoidable in early pregnancy, termination may be discussed within a non-directive, multidisciplinary framework. Patient autonomy, values, and psychosocial support are central to this decision-making process.

## 5. Conclusions

This review underscores the rarity and complexity of neuroendocrine neoplasms in pregnancy, highlighting a critical intersection between oncology, obstetrics, and individualized care. The lack of robust evidence for most diagnostic and treatment modalities in this population necessitates a paradigm grounded in personalized medicine.

The diagnosis and management of NENs during pregnancy present complex and multifaceted challenges. These arise primarily due to the rarity of such cases; the nonspecific nature of presenting symptoms, which may overlap with normal physiological changes in pregnancy; and the critical need for multidisciplinary coordination to ensure the best possible outcomes for both mother and fetus. WB-DWI/MRI is the imaging modality of choice for oncologic staging during pregnancy, offering high diagnostic accuracy while minimizing fetal risk, thereby supporting more informed and effective treatment planning. In the absence of large-scale studies or randomized clinical trials, current clinical practice is largely informed by case reports, retrospective series, and expert consensus, highlighting a significant gap in high-quality evidence.

Optimal care requires the collaboration of multiple specialties, including endocrinology, obstetrics and gynecology, medical and surgical oncology, anesthesiology, and neonatology. This interdisciplinary approach is vital for devising individualized treatment plans that address not only the oncologic characteristics of the tumor but also the gestational context. Key clinical considerations include the selection and timing of medical therapies, particularly those with potential teratogenic effects; the scheduling of surgical interventions with minimal risk to the fetus; decisions regarding the mode and timing of delivery; and the development of anesthesia protocols that prioritize both maternal safety and fetal well-being.

Pregnancy termination may be necessary, particularly during the first trimester, due to the high teratogenic risk of treatment. Decisions are complex and depend on tumor characteristics, gestational age, treatment urgency, and patient preferences. Surgery can be performed at any gestational stage but must be carefully individualized. Systemic therapies are generally not recommended during pregnancy due to limited safety data and potential fetal risks. The use of SSAs, while occasionally reported as safe—primarily in cases of acromegaly—remains uncertain during pregnancy.

By tailoring oncologic interventions to the molecular characteristics of the tumor, gestational age, and patient priorities, clinicians can optimize outcomes despite limited data. This patient-centered, evidence-informed approach embodies the future of cancer care in pregnancy—one that transcends rigid protocols to account for the nuances of each case.

Given these complexities, there is a pressing need for continued research to improve our understanding of NENs in pregnancy and to develop evidence-based guidelines. Future studies should aim to clarify the safety profiles of systemic therapies, explore the long-term outcomes for exposed offspring, and establish standardized protocols for managing both localized and metastatic disease during gestation. Advancing this field will allow healthcare professionals to make more informed, confident decisions and provide comprehensive care to pregnant patients with NENs, ultimately improving maternal and neonatal outcomes.

## Figures and Tables

**Table 1:** NEN related hereditary syndromes and pregnancy.

Hereditary Syndrome	Genetic Mutation	Clinical Characteristics	Epidemiological Characteristics	Relation to Pregnancy
MEN1 [24,25,26,27,28,29]	*MEN1* gene (menin, tumor suppressor)	Multifocal endocrine tumors affecting parathyroid, pancreas, pituitary, and thymus/bronchial tubes.	Prevalence: 1/20,000–1/40,000. High penetrance, equal sex distribution. Primary hyperparathyroidism in >90% of cases. pNET in 30–80% of patients by age 50.	Rare during pregnancy.
MEN2 [30]	*RET* proto-oncogene	Medullary thyroid carcinoma, pheochromocytoma, and hyperparathyroidism. NETs less common but possible.	Prevalence: 1/35,000. MTC in 25–30% of hereditary cases, onset at 30–40 years (earlier than sporadic). High penetrance.	Pheochromocytoma poses significant risk (hypertensive crisis).
VHL [31]	*VHL* gene (tumor suppressor)	pNET, pheochromocytomas, hemangioblastomas, renal cell carcinoma.	Prevalence: 1/36,000. 90% penetrance by age 65. pNET in 10–17% of cases.	pNET and pheochromocytomas rare in pregnancy but reported.
NF1 [32,33]	*NF1* gene (neurofibromin, RAS regulator)	Neurofibromas, optic pathway gliomas, and rare NET.	Prevalence: 1/2500–1/3500. NETs in <5% of cases. Variable expressivity.	NET extremely rare in pregnancy.
TSC [34]	*TSC1* (hamartin) or *TSC2* (tuberin)	Benign hamartomas, low-grade pancreatic NET (1–5% malignant islet cell tumors).	Prevalence: 1/6000–1/10,000. NET rare (<5%).	NET in pregnancy are very rare.
MEN4 [35]	*CDKN1B* gene (p27, cell cycle regulator)	NETs in pituitary, parathyroid, and pancreas, similar to MEN1 but rarer. Well-differentiated tumors.	Prevalence: Very rare (only 19 reported cases). Onset typically <30 years.	No specific pregnancy data; likely similar to MEN1.

MEN—multiple endocrine neoplasia; VHL—von Hippel–Lindau; NF1—neurofibromatosis type 1; TSC—tuberous sclerosis complex; pNET—pancreatic neuroendocrine tumor; MTC—medullary thyroid carcinoma; NET—neuroendocrine tumor.

**Table 2:** Recommendations for use during pregnancy according to FDA and EMA.

Drug	FDA	EMA	Drug	FDA	EMA
Lanreotide	Category not assigned; use if benefit justifies risk [87]	Not recommended; use only if the benefit justifies the risk [88]	Cabozantinib	Category not assigned; use not recommended [89]	Contraindicated due to reproductive toxicity [90]
Octreotide	Category B (previous); no evidence of risk to humans [91]	Not recommended; use only if the benefit justifies the risk [92]	Capecitabine	Category not assigned; use contraindicated [93]	Contraindicated due to reproductive toxicity [94]
Everolimus	Category not assigned; use not recommended due to potential risk [95]	Contraindicated due to reproductive toxicity [96]	Temozolomide	Category D: evidence of fetal risk [97]	Contraindicated due to reproductive toxicity [98]
Sunitinib	Category D: evidence of fetal risk [99]	Contraindicated due to reproductive toxicity [100]	177Lu- DOTATATE	Category not assigned; use contraindicated [101]	Contraindicated due to ionizing radiation [102]

FDA—Food and Drug Administration; EMA—European Medicines Agency; Lu—lutetium.

## Data Availability

The original contributions presented in this study are included in the article. Further inquiries can be directed to the corresponding author(s).

## Abbreviations

The following abbreviations are used in this manuscript:

NEN	Neuroendocrine Neoplasms
ICU	Intensive Care Unit
NET	Neuroendocrine Tumor
NEC	Neuroendocrine Carcinoma
MiNEN	Mixed Neuroendocrine–Non-Neuroendocrine Neoplasm
APC	Annual Percentage Change
SEER	Surveillance, Epidemiology, and End Results
MEN	Multiple Endocrine Neoplasia
VHL	von Hippel–Lindau
NF1	Neurofibromatosis Type 1
TSC	Tuberous Sclerosis Complex
pNET	Pancreatic Neuroendocrine Tumor
MTC	Medullary Thyroid Carcinoma
AOR	Adjusted Odds Ratio
TNENs	Thymic Neuroendocrine Neoplasms
ACTH	Adrenocorticotropic Hormone
PET-CT	Positron Emission Tomography–Computed Tomography
SSA	Somatostatin Analogs
WB-DWI/MRI	Whole-Body Diffusion-Weighted MRI
CgA	Chromogranin A
5-HIAA	5-Hydroxyindoleacetic Acid
ENETS	European Neuroendocrine Tumor Society
TACE	Transcatheter Arterial Chemoembolization
RT	Radiotherapy
PRRT	Peptide Receptor Radionuclide Therapy
FDA	Food and Drug Administration
EMA	European Medicines Agency
Lu	Lutetium
